# Publisher Correction: Brain charts for the human lifespan

**DOI:** 10.1038/s41586-022-05300-0

**Published:** 2022-09-23

**Authors:** R. A. I. Bethlehem, J. Seidlitz, S. R. White, J. W. Vogel, K. M. Anderson, C. Adamson, S. Adler, G. S. Alexopoulos, E. Anagnostou, A. Areces-Gonzalez, D. E. Astle, B. Auyeung, M. Ayub, J. Bae, G. Ball, S. Baron-Cohen, R. Beare, S. A. Bedford, V. Benegal, F. Beyer, J. Blangero, M. Blesa Cábez, J. P. Boardman, M. Borzage, J. F. Bosch-Bayard, N. Bourke, V. D. Calhoun, M. M. Chakravarty, C. Chen, C. Chertavian, G. Chetelat, Y. S. Chong, J. H. Cole, A. Corvin, M. Costantino, E. Courchesne, F. Crivello, V. L. Cropley, J. Crosbie, N. Crossley, M. Delarue, R. Delorme, S. Desrivieres, G. A. Devenyi, M. A. Di Biase, R. Dolan, K. A. Donald, G. Donohoe, K. Dunlop, A. D. Edwards, J. T. Elison, C. T. Ellis, J. A. Elman, L. Eyler, D. A. Fair, E. Feczko, P. C. Fletcher, P. Fonagy, C. E. Franz, L. Galan-Garcia, A. Gholipour, J. Giedd, J. H. Gilmore, D. C. Glahn, I. M. Goodyer, P. E. Grant, N. A. Groenewold, F. M. Gunning, R. E. Gur, R. C. Gur, C. F. Hammill, O. Hansson, T. Hedden, A. Heinz, R. N. Henson, K. Heuer, J. Hoare, B. Holla, A. J. Holmes, R. Holt, H. Huang, K. Im, J. Ipser, C. R. Jack, A. P. Jackowski, T. Jia, K. A. Johnson, P. B. Jones, D. T. Jones, R. S. Kahn, H. Karlsson, L. Karlsson, R. Kawashima, E. A. Kelley, S. Kern, K. W. Kim, M. G. Kitzbichler, W. S. Kremen, F. Lalonde, B. Landeau, S. Lee, J. Lerch, J. D. Lewis, J. Li, W. Liao, C. Liston, M. V. Lombardo, J. Lv, C. Lynch, T. T. Mallard, M. Marcelis, R. D. Markello, S. R. Mathias, B. Mazoyer, P. McGuire, M. J. Meaney, A. Mechelli, N. Medic, B. Misic, S. E. Morgan, D. Mothersill, J. Nigg, M. Q. W. Ong, C. Ortinau, R. Ossenkoppele, M. Ouyang, L. Palaniyappan, L. Paly, P. M. Pan, C. Pantelis, M. M. Park, T. Paus, Z. Pausova, D. Paz-Linares, A. Pichet Binette, K. Pierce, X. Qian, J. Qiu, A. Qiu, A. Raznahan, T. Rittman, A. Rodrigue, C. K. Rollins, R. Romero-Garcia, L. Ronan, M. D. Rosenberg, D. H. Rowitch, G. A. Salum, T. D. Satterthwaite, H. L. Schaare, R. J. Schachar, A. P. Schultz, G. Schumann, M. Schöll, D. Sharp, R. T. Shinohara, I. Skoog, C. D. Smyser, R. A. Sperling, D. J. Stein, A. Stolicyn, J. Suckling, G. Sullivan, Y. Taki, B. Thyreau, R. Toro, N. Traut, K. A. Tsvetanov, N. B. Turk-Browne, J. J. Tuulari, C. Tzourio, É. Vachon-Presseau, M. J. Valdes-Sosa, P. A. Valdes-Sosa, S. L. Valk, T. van Amelsvoort, S. N. Vandekar, L. Vasung, L. W. Victoria, S. Villeneuve, A. Villringer, P. E. Vértes, K. Wagstyl, Y. S. Wang, S. K. Warfield, V. Warrier, E. Westman, M. L. Westwater, H. C. Whalley, A. V. Witte, N. Yang, B. Yeo, H. Yun, A. Zalesky, H. J. Zar, A. Zettergren, J. H. Zhou, H. Ziauddeen, A. Zugman, X. N. Zuo, 3R-BRAIN, AIBL, C. Rowe, Alzheimer’s Disease Neuroimaging Initiative, Alzheimer’s Disease Repository Without Borders Investigators, G. B. Frisoni, CALM Team, Cam-CAN, CCNP, COBRE, cVEDA, ENIGMA Developmental Brain Age Working Group, Developing Human Connectome Project, FinnBrain, Harvard Aging Brain Study, IMAGEN, KNE96, The Mayo Clinic Study of Aging, NSPN, POND, The PREVENT-AD Research Group, A. Pichet Binette, VETSA, E. T. Bullmore, A. F. Alexander-Bloch

**Affiliations:** 1 Autism Research Centre, Department of Psychiatry, University of Cambridge, Cambridge, UK; 2 Brain Mapping Unit, Department of Psychiatry, University of Cambridge, Cambridge, UK; 3 Department of Psychiatry, University of Pennsylvania, Philadelphia, PA USA; 4 Department of Child and Adolescent Psychiatry and Behavioral Science, The Children’s Hospital of Philadelphia, Philadelphia, PA USA; 5 Lifespan Brain Institute, The Children’s Hospital of Philadelphia and Penn Medicine, Philadelphia, PA USA; 6 Department of Psychiatry, University of Cambridge, Cambridge, UK; 7 MRC Biostatistics Unit, University of Cambridge, Cambridge, UK; 8 Lifespan Informatics & Neuroimaging Center, University of Pennsylvania, Philadelphia, PA USA; 9 Department of Psychology, Yale University, New Haven, CT USA; 10 Developmental Imaging, Murdoch Children’s Research Institute, Melbourne, Victoria Australia; 11 Department of Medicine, Monash University, Melbourne, Victoria Australia; 12 UCL Great Ormond Street Institute for Child Health, London, UK; 13 Weill Cornell Institute of Geriatric Psychiatry, Department of Psychiatry, Weill Cornell Medicine, New York, USA; 14 Department of Pediatrics University of Toronto, Toronto, Canada; 15 Holland Bloorview Kids Rehabilitation Hospital, Toronto, Canada; 16 The Clinical Hospital of Chengdu Brain Science Institute, MOE Key Lab for NeuroInformation, University of Electronic Science and Technology of China, Chengdu, China; 17 University of Pinar del Río “Hermanos Saiz Montes de Oca”, Pinar del Río, Cuba; 18 MRC Cognition and Brain Sciences Unit, University of Cambridge, Cambridge, UK; 19 Department of Psychology, School of Philosophy, Psychology and Language Sciences, University of Edinburgh, Edinburgh, UK; 20 Queen’s University, Department of Psychiatry, Centre for Neuroscience Studies, Kingston, Ontario Canada; 21 University College London, Mental Health Neuroscience Research Department, Division of Psychiatry, London, UK; 22 Department of Neuropsychiatry, Seoul National University Bundang Hospital, Seongnam, Korea; 23 Department of Paediatrics, University of Melbourne, Melbourne, Victoria Australia; 24 Cambridge Lifetime Asperger Syndrome Service (CLASS), Cambridgeshire and Peterborough NHS Foundation Trust, Cambridge, UK; 25 Centre for Addiction Medicine, National Institute of Mental Health and Neurosciences (NIMHANS), Bengaluru, India; 26 Department of Neurology, Max Planck Institute for Human Cognitive and Brain Sciences, Leipzig, Germany; 27 Department of Human Genetics, South Texas Diabetes and Obesity Institute, University of Texas Rio Grande Valley, Edinburg, TX USA; 28 MRC Centre for Reproductive Health, University of Edinburgh, Edinburgh, UK; 29 Fetal and Neonatal Institute, Division of Neonatology, Children’s Hospital Los Angeles, Department of Pediatrics, Keck School of Medicine, University of Southern California, Los Angeles, CA USA; 30 McGill Centre for Integrative Neuroscience, Ludmer Centre for Neuroinformatics and Mental Health, Montreal Neurological Institute, Montreal, Quebec Canada; 31 McGill University, Montreal, Quebec Canada; 32 Department of Brain Sciences, Imperial College London, London, UK; 33 Care Research and Technology Centre, Dementia Research Institute, London, UK; 34 Tri-institutional Center for Translational Research in Neuroimaging and Data Science (TReNDS), Georgia State University, Georgia Institute of Technology, and Emory University, Atlanta, GA USA; 35 Computational Brain Anatomy (CoBrA) Laboratory, Cerebral Imaging Centre, Douglas Mental Health University Institute, Montreal, Quebec Canada; 36 Penn Statistics in Imaging and Visualization Center, Department of Biostatistics, Epidemiology, and Informatics, Perelman School of Medicine, University of Pennsylvania, Philadelphia, PA USA; 37 Normandie Univ, UNICAEN, INSERM, U1237, PhIND “Physiopathology and Imaging of Neurological Disorders”, Institut Blood and Brain @ Caen-Normandie, Cyceron, Caen, France; 38 Singapore Institute for Clinical Sciences, Agency for Science, Technology and Research, Singapore, Singapore; 39 Department of Obstetrics and Gynaecology, Yong Loo Lin School of Medicine, National University of Singapore, Singapore, Singapore; 40 Centre for Medical Image Computing (CMIC), University College London, London, UK; 41 Dementia Research Centre (DRC), University College London, London, UK; 42 Department of Psychiatry, Trinity College, Dublin, Ireland; 43 Cerebral Imaging Centre, Douglas Mental Health University Institute, Verdun, Quebec Canada; 44 Undergraduate program in Neuroscience, McGill University, Montreal, Quebec Canada; 45 Department of Neuroscience, University of California, San Diego, San Diego, CA USA; 46 Autism Center of Excellence, University of California, San Diego, San Diego, CA USA; 47 Institute of Neurodegenerative Disorders, CNRS UMR5293, CEA, University of Bordeaux, Bordeaux, France; 48 Melbourne Neuropsychiatry Centre, University of Melbourne, Melbourne, Victoria Australia; 49 The Hospital for Sick Children, Toronto, Ontario Canada; 50 Department of Psychiatry, School of Medicine, Pontificia Universidad Católica de Chile, Santiago, Chile; 51 Department of Psychosis Studies, Institute of Psychiatry, Psychology and Neuroscience, King’s College London, London, UK; 52 Instituto Milenio Intelligent Healthcare Engineering, Santiago, Chile; 53 Child and Adolescent Psychiatry Department, Robert Debré University Hospital, AP-HP, Paris, France; 54 Human Genetics and Cognitive Functions, Institut Pasteur, Paris, France; 55 Social, Genetic and Developmental Psychiatry Centre, Institute of Psychiatry, Psychology and Neuroscience, King’s College London, London, UK; 56 Cerebral Imaging Centre, McGill Department of Psychiatry, Douglas Mental Health University Institute, Montreal, QC Canada; 57 Department of Psychiatry, McGill University, Montreal, QC Canada; 58 Department of Psychiatry, Brigham and Women’s Hospital, Harvard Medical School, Boston, MA USA; 59 Max Planck UCL Centre for Computational Psychiatry and Ageing Research, University College London, London, UK; 60 Wellcome Centre for Human Neuroimaging, London, UK; 61 Division of Developmental Paediatrics, Department of Paediatrics and Child Health, Red Cross War Memorial Children’s Hospital, Cape Town, South Africa; 62 Neuroscience Institute, University of Cape Town, Cape Town, South Africa; 63 Center for Neuroimaging, Cognition & Genomics (NICOG), School of Psychology, National University of Ireland Galway, Galway, Ireland; 64 Weil Family Brain and Mind Research Institute, Department of Psychiatry, Weill Cornell Medicine, New York, NY USA; 65 Centre for the Developing Brain, King’s College London, London, UK; 66 Evelina London Children’s Hospital, London, UK; 67 MRC Centre for Neurodevelopmental Disorders, London, UK; 68 Institute of Child Development, Department of Pediatrics, Masonic Institute for the Developing Brain, University of Minnesota, Minneapolis, MN USA; 69 Haskins Laboratories, New Haven, CT USA; 70 Department of Psychiatry, Center for Behavior Genetics of Aging, University of California, San Diego, La Jolla, CA USA; 71 Desert-Pacific Mental Illness Research Education and Clinical Center, VA San Diego Healthcare, San Diego, CA USA; 72 Department of Psychiatry, University of California San Diego, Los Angeles, CA USA; 73 Department of Psychiatry, University of Cambridge, and Wellcome Trust MRC Institute of Metabolic Science, Cambridge Biomedical Campus, Cambridge, UK; 74 Cambridgeshire and Peterborough NHS Foundation Trust, Cambridge, UK; 75 Department of Clinical, Educational and Health Psychology, University College London, London, UK; 76 Anna Freud National Centre for Children and Families, London, UK; 77 Cuban Center for Neuroscience, La Habana, Cuba; 78 Computational Radiology Laboratory, Boston Children’s Hospital, Boston, MA USA; 79 Department of Child and Adolescent Psychiatry, University of California, San Diego, San Diego, CA USA; 80 Department of Psychiatry, University of California San Diego, San Diego, CA USA; 81 Department of Psychiatry, University of North Carolina, Chapel Hill, NC USA; 82 Department of Psychiatry, Boston Children’s Hospital and Harvard Medical School, Boston, MA USA; 83 Harvard Medical School, Boston, MA USA; 84 Division of Newborn Medicine and Neuroradiology, Fetal Neonatal Neuroimaging and Developmental Science Center, Boston Children’s Hospital, Harvard Medical School, Boston, MA USA; 85 Department of Paediatrics and Child Health, Red Cross War Memorial Children’s Hospital, SA-MRC Unit on Child & Adolescent Health, University of Cape Town, Cape Town, South Africa; 86 Weill Cornell Institute of Geriatric Psychiatry, Department of Psychiatry, Weill Cornell Medicine, New York, NY USA; 87 Mouse Imaging Centre, Toronto, Ontario Canada; 88 Clinical Memory Research Unit, Department of Clinical Sciences Malmö, Lund University, Malmö, Sweden; 89 Memory Clinic, Skåne University Hospital, Malmö, Sweden; 90 Department of Neurology, Icahn School of Medicine at Mount Sinai, New York, NY USA; 91 Athinoula A. Martinos Center for Biomedical Imaging, Department of Radiology, Massachusetts General Hospital, Harvard Medical School, Boston, MA USA; 92 Charité – Universitätsmedizin Berlin, corporate member of Freie Universität Berlin and Humboldt-Universität zu Berlin, Department of Psychiatry and Psychotherapy, Charité Campus Mitte, Berlin, Germany; 93 Department of Neuropsychology, Max Planck Institute for Human Cognitive and Brain Sciences, Leipzig, Germany; 94 Université de Paris, Paris, France; 95 Department of Psychiatry, University of Cape Town, Cape Town, South Africa; 96 Department of Integrative Medicine, NIMHANS, Bengaluru, India; 97 Accelerator Program for Discovery in Brain disorders using Stem cells (ADBS), Department of Psychiatry, NIMHANS, Bengaluru, India; 98 Departments of Psychology and Psychiatry, Yale University, New Haven, CT USA; 99 Radiology Research, Children’s Hospital of Philadelphia, Philadelphia, PA USA; 100 The Department of Radiology, Perelman School of Medicine, University of Pennsylvania, Philadelphia, PA USA; 101 Department of Psychiatry and Mental Health, Clinical Neuroscience Institute, University of Cape Town, Cape Town, South Africa; 102 Department of Radiology, Mayo Clinic, Rochester, MN USA; 103 Department of Psychiatry, Universidade Federal de São Paulo, São Paulo, Brazil; 104 National Institute of Developmental Psychiatry, Beijing, China; 105 Institute of Science and Technology for Brain-Inspired Intelligence, Fudan University, Shanghai, China; 106 Key Laboratory of Computational Neuroscience and BrainInspired Intelligence (Fudan University), Ministry of Education, Shanghai, China; 107 Centre for Population Neuroscience and Precision Medicine (PONS), Institute of Psychiatry, Psychology and Neuroscience, SGDP Centre, King’s College London, London, UK; 108 Harvard Aging Brain Study, Department of Neurology, Massachusetts General Hospital, Boston, MA USA; 109 Center for Alzheimer Research and Treatment, Department of Neurology, Brigham and Women’s Hospital, Boston, MA USA; 110 Department of Radiology, Massachusetts General Hospital, Boston, MA USA; 111 Department of Neurology, Mayo Clinic, Rochester, MN USA; 112 Department of Psychiatry, Icahn School of Medicine, Mount Sinai, NY USA; 113 Department of Clinical Medicine, Department of Psychiatry and Turku Brain and Mind Center, FinnBrain Birth Cohort Study, University of Turku and Turku University Hospital, Turku, Finland; 114 Centre for Population Health Research, Turku University Hospital and University of Turku, Turku, Finland; 115 Institute of Development, Aging and Cancer, Tohoku University, Seiryocho, Aobaku, Sendai, Japan; 116 Queen’s University, Departments of Psychology and Psychiatry, Centre for Neuroscience Studies, Kingston, Ontario Canada; 117 Neuropsychiatric Epidemiology Unit, Department of Psychiatry and Neurochemistry, Institute of Neuroscience and Physiology, the Sahlgrenska Academy, Centre for Ageing and Health (AGECAP) at the University of Gothenburg, Gothenburg, Sweden; 118 Region Västra Götaland, Sahlgrenska University Hospital, Psychiatry, Cognition and Old Age Psychiatry Clinic, Gothenburg, Sweden; 119 Department of Brain and Cognitive Sciences, Seoul National University College of Natural Sciences, Seoul, South Korea; 120 Department of Neuropsychiatry, Seoul National University Bundang Hospital, Seongnam, South Korea; 121 Department of Psychiatry, Seoul National University College of Medicine, Seoul, South Korea; 122 Institute of Human Behavioral Medicine, SNU-MRC, Seoul, South Korea; 123 Section on Developmental Neurogenomics, Human Genetics Branch, National Institute of Mental Health, Bethesda, MD USA; 124 Department of Brain & Cognitive Sciences, Seoul National University College of Natural Sciences, Seoul, South Korea; 125 Department of Medical Biophysics, University of Toronto, Toronto, Ontario Canada; 126 Wellcome Centre for Integrative Neuroimaging, FMRIB, Nuffield Department of Clinical Neuroscience, University of Oxford, Oxford, UK; 127 Montreal Neurological Institute, McGill University, Montreal, Quebec Canada; 128 The Clinical Hospital of Chengdu Brain Science Institute, University of Electronic Science and Technology of China, Chengdu, China; 129 Department of Psychiatry and Brain and Mind Research Institute, Weill Cornell Medicine, New York, NY USA; 130 Laboratory for Autism and Neurodevelopmental Disorders, Center for Neuroscience and Cognitive Systems @UniTn, Istituto Italiano di Tecnologia, Rovereto, Italy; 131 School of Biomedical Engineering and Brain and Mind Centre, The University of Sydney, Sydney, New South Wales Australia; 132 Department of Psychology, University of Texas, Austin, TX USA; 133 Department of Psychiatry and Neuropsychology, School of Mental Health and Neuroscience, EURON, Maastricht University Medical Centre, Maastricht, The Netherlands; 134 Institute for Mental Health Care Eindhoven (GGzE), Eindhoven, The Netherlands; 135 McConnell Brain Imaging Centre, Montreal Neurological Institute, McGill University, Montreal, Quebec Canada; 136 Ludmer Centre for Neuroinformatics and Mental Health, Douglas Mental Health University Institute, Montreal, Quebec Canada; 137 Singapore Institute for Clinical Sciences, Singapore, Singapore; 138 Bordeaux University Hospital, Bordeaux, France; 139 Department of Computer Science and Technology, University of Cambridge, Cambridge, UK; 140 The Alan Turing Institute, London, UK; 141 Department of Psychology, School of Business, National College of Ireland, Dublin, Ireland; 142 School of Psychology and Center for Neuroimaging and Cognitive Genomics, National University of Ireland Galway, Galway, Ireland; 143 Department of Psychiatry, Trinity College Dublin, Dublin, Ireland; 144 Department of Psychiatry, School of Medicine, Oregon Health and Science University, Portland, OR USA; 145 Center for Sleep and Cognition, Yong Loo Lin School of Medicine, National University of Singapore, Singapore, Singapore; 146 Department of Pediatrics, Washington University in St Louis, St Louis, MO USA; 147 Alzheimer Center Amsterdam, Department of Neurology, Amsterdam Neuroscience, Vrije Universiteit Amsterdam, Amsterdam UMC, Amsterdam, The Netherlands; 148 Lund University, Clinical Memory Research Unit, Lund, Sweden; 149 Robarts Research Institute and The Brain and Mind Institute, University of Western Ontario, London, Ontario Canada; 150 Department of Psychiatry, Federal University of Sao Poalo (UNIFESP), Sao Poalo, Brazil; 151 National Institute of Developmental Psychiatry for Children and Adolescents (INPD), Sao Poalo, Brazil; 152 Melbourne Neuropsychiatry Centre, Department of Psychiatry, The University of Melbourne and Melbourne Health, Carlton South, Victoria Australia; 153 Melbourne School of Engineering, The University of Melbourne, Parkville, Victoria Australia; 154 Florey Institute of Neuroscience and Mental Health, Parkville, Victoria Australia; 155 Department of Psychiatry, Schulich School of Medicine and Dentistry, Western University, London, Ontario Canada; 156 Department of Psychiatry, Faculty of Medicine and Centre Hospitalier Universitaire Sainte-Justine, University of Montreal, Montreal, Quebec Canada; 157 Departments of Psychiatry and Psychology, University of Toronto, Toronto, Ontario Canada; 158 Departments of Physiology and Nutritional Sciences, University of Toronto, Toronto, Ontario Canada; 159 Cuban Neuroscience Center, Havana, Cuba; 160 Department of Psychiatry, Faculty of Medicine, McGill University, Montreal, Quebec Canada; 161 Douglas Mental Health University Institute, Montreal, Quebec Canada; 162 School of Psychology, Southwest University, Chongqing, China; 163 Department of Biomedical Engineering, The N.1 Institute for Health, National University of Singapore, Singapore, Singapore; 164 Department of Clinical Neurosciences, University of Cambridge, Cambridge, UK; 165 Department of Neurology, Harvard Medical School, Boston, MA USA; 166 Department of Neurology, Boston Children’s Hospital, Boston, MA USA; 167 Instituto de Biomedicina de Sevilla (IBiS) HUVR/CSIC/Universidad de Sevilla, Dpto. de Fisiología Médica y Biofísica, Seville, Spain; 168 Department of Psychology and Neuroscience Institute, University of Chicago, Chicago, IL USA; 169 Department of Paediatrics and Wellcome-MRC Cambridge Stem Cell Institute, University of Cambridge, Cambridge, UK; 170 Department of Psychiatry, Universidade Federal do Rio Grande do Sul (UFRGS), Hospital de Clinicas de Porto Alegre, Porto Alegre, Brazil; 171 National Institute of Developmental Psychiatry (INPD), São Paulo, Brazil; 172 Otto Hahn Group Cognitive Neurogenetics, Max Planck Institute for Human Cognitive and Brain Sciences, Leipzig, Germany; 173 Institute of Neuroscience and Medicine (INM-7: Brain and Behaviour), Research Centre Juelich, Juelich, Germany; 174 Athinoula A. Martinos Center for Biomedical Imaging, Department of Radiology, Massachusetts General Hospital, Charlestown, MA USA; 175 Centre for Population Neuroscience and Stratified Medicine (PONS), Institute for Science and Technology for Brain-inspired Intelligence, Fudan University, Shanghai, China; 176 PONS-Centre, Charite Mental Health, Dept of Psychiatry and Psychotherapy, Charite Campus Mitte, Berlin, Germany; 177 Wallenberg Centre for Molecular and Translational Medicine, University of Gothenburg, Gothenburg, Sweden; 178 Department of Psychiatry and Neurochemistry, University of Gothenburg, Gothenburg, Sweden; 179 Dementia Research Centre, Queen’s Square Institute of Neurology, University College London, London, UK; 180 Care Research and Technology Centre, UK Dementia Research Institute, London, UK; 181 Center for Biomedical Image Computing and Analytics, Department of Radiology, Perelman School of Medicine, University of Pennsylvania, Philadelphia, PA USA; 182 Departments of Neurology, Pediatrics, and Radiology, Washington University School of Medicine, St Louis, MO USA; 183 SA MRC Unit on Risk and Resilience in Mental Disorders, Dept of Psychiatry and Neuroscience Institute, University of Cape Town, Cape Town, South Africa; 184 Division of Psychiatry, Centre for Clinical Brain Sciences, University of Edinburgh, Edinburgh, UK; 185 Department of Neuroscience, Institut Pasteur, Paris, France; 186 Center for Research and Interdisciplinarity (CRI), Université Paris Descartes, Paris, France; 187 Department of Psychology, University of Cambridge, Cambridge, UK; 188 Wu Tsai Institute, Yale University, New Haven, CT USA; 189 Department of Clinical Medicine, University of Turku, Turku, Finland; 190 Turku Collegium for Science, Medicine and Technology, University of Turku, Turku, Finland; 191 Univ. Bordeaux, Inserm, Bordeaux Population Health Research Center, U1219, CHU Bordeaux, Bordeaux, France; 192 Faculty of Dental Medicine and Oral Health Sciences, McGill University, Montreal, Quebec Canada; 193 Alan Edwards Centre for Research on Pain (AECRP), McGill University, Montreal, Quebec Canada; 194 Institute for Neuroscience and Medicine 7, Forschungszentrum Jülich, Jülich, Germany; 195 Max Planck Institute for Human Cognitive and Brain Sciences, Leipzig, Germany; 196 Department of Psychiatry and Neurosychology, Maastricht University, Maastricht, The Netherlands; 197 Department of Biostatistics, Vanderbilt University, Nashville, TN USA; 198 Department of Biostatistics, Vanderbilt University Medical Center, Nashville, TN USA; 199 Clinic for Cognitive Neurology, University of Leipzig Medical Center, Leipzig, Germany; 200 State Key Laboratory of Cognitive Neuroscience and Learning, Beijing Normal University, Beijing, China; 201 Developmental Population Neuroscience Research Center, IDG/McGovern Institute for Brain Research, Beijing Normal University, Beijing, China; 202 National Basic Science Data Center, Beijing, China; 203 Research Center for Lifespan Development of Brain and Mind, Institute of Psychology, Chinese Academy of Sciences, Beijing, China; 204 Division of Clinical Geriatrics, Center for Alzheimer Research, Department of Neurobiology, Care Sciences and Society, Karolinska Institutet, Stockholm, Sweden; 205 Faculty of Medicine, CRC 1052 ‘Obesity Mechanisms’, University of Leipzig, Leipzig, Germany; 206 Department of Electrical and Computer Engineering, National University of Singapore, Singapore, Singapore; 207 Centre for Sleep and Cognition and Centre for Translational MR Research, Yong Loo Lin School of Medicine, National University of Singapore, Singapore, Singapore; 208 N.1 Institute for Health & Institute for Digital Medicine, National University of Singapore, Singapore, Singapore; 209 Integrative Sciences and Engineering Programme (ISEP), National University of Singapore, Singapore, Singapore; 210 Department of Biomedical Engineering, University of Melbourne, Melbourne, Victoria Australia; 211 Center for Translational Magnetic Resonance Research, Yong Loo Lin School of Medicine, National University of Singapore, Singapore, Singapore; 212 Wellcome Trust-MRC Institute of Metabolic Science, University of Cambridge, Cambridge, UK; 213 National Institute of Mental Health (NIMH), National Institutes of Health (NIH), Bethesda, MD USA; 214 Department of Psychiatry, Escola Paulista de Medicina, São Paulo, Brazil; 215 Key Laboratory of Brain and Education, School of Education Science, Nanning Normal University, Nanning, China; 216 Memory Disorders Clinic, Austin Health, Melbourne, Victoria Australia; 217 University Hospitals and University of Geneva, Geneva, Switzerland; 218 IRCCS Fatebenefratelli, The National Centre for Alzheimer’s and Mental Diseases, Brescia, Italy

**Keywords:** Neural ageing, Diseases of the nervous system, Cognitive neuroscience, Development of the nervous system

Correction to: *Nature* 10.1038/s41586-022-04554-y Published online 6 April 2022

In the version of this article initially published, there were errors in the affiliations for K. Im (missing affiliation, Division of Newborn Medicine and Neuroradiology, Fetal Neonatal Neuroimaging and Developmental Science Center, Boston Children’s Hospital, Harvard Medical School, Boston, MA, USA), J. Lerch (missing affiliation, Mouse Imaging Centre, Toronto, Ontario, Canada), S. Villeneuve and X. N. Zuo (incorrect affiliation numbers listed), H. Yun (missing affiliation, Division of Newborn Medicine and Neuroradiology, Fetal Neonatal Neuroimaging and Developmental Science Center, Boston Children’s Hospital, Harvard Medical School, Boston, MA, USA), and H. J. Zar (extra affiliation shown). In addition, the affiliation numbers for all authors listed in the consortium membership section were incorrect by 1–3 digits. The errors have been corrected in the HTML and PDF versions of the article.

