# Supplementary material for: Brain charts for the human lifespan
Source: Nature. 2022 Apr 6;604(7906):525–33. doi: 10.1038/s41586-022-04554-y (PMC9021021; doi:10.1038/s41586-022-04554-y)
Supplement: Supplementary file 2 — Reporting Summary [file 41586_2022_4554_MOESM2_ESM.pdf]

nature portfolio

Corresponding author(s): Richard A.I. Bethlehem, Jakob Seidlitz

Last updated by author(s): Feb 2, 2022

Reporting Summary

Nature Portfolio wishes to improve the reproducibility of the work that we publish. This form provides structure for consistency and transparency in reporting. For further information on Nature Portfolio policies, see our Editorial Policies and the Editorial Policy Checklist.

Statistics

For all statistical analyses, confirm that the following items are present in the figure legend, table legend, main text, or Methods section.

n/a	Confirmed	
☐	☒	The exact sample size (*n*) for each experimental group/condition, given as a discrete number and unit of measurement
☐	☒	A statement on whether measurements were taken from distinct samples or whether the same sample was measured repeatedly
☐	☒	The statistical test(s) used AND whether they are one- or two-sided<br>*Only common tests should be described solely by name; describe more complex techniques in the Methods section.*
☐	☒	A description of all covariates tested
☐	☒	A description of any assumptions or corrections, such as tests of normality and adjustment for multiple comparisons
☐	☒	A full description of the statistical parameters including central tendency (e.g. means) or other basic estimates (e.g. regression coefficient) AND variation (e.g. standard deviation) or associated estimates of uncertainty (e.g. confidence intervals)
☐	☒	For null hypothesis testing, the test statistic (e.g. *F*, *t*, *r*) with confidence intervals, effect sizes, degrees of freedom and *P* value noted<br>*Give P values as exact values whenever suitable.*
☐	☒	For Bayesian analysis, information on the choice of priors and Markov chain Monte Carlo settings
☐	☒	For hierarchical and complex designs, identification of the appropriate level for tests and full reporting of outcomes
☐	☒	Estimates of effect sizes (e.g. Cohen's *d*, Pearson's *r*), indicating how they were calculated

Our web collection on statistics for biologists contains articles on many of the points above.

Software and code

Policy information about availability of computer code

Data collection	No software was used in data collection
Data analysis	Data was analysed using a combination of open source R code (v4.1.2) and custom R code made available on https://github.com/ucam-department-of-psychiatry/Lifespan. With respect to all visualisation and statistics represented in graphical format, unless otherwise stated these were generated in R GNU v4.1.2 using the “ggplot” package. Where boxplots are used they indicate the median and lower and upper hinges correspond to the first and third quartiles (the 25th and 75th percentiles). The upper whisker extends from the hinge to the largest value no further than 1.5 * IQR from the hinge (where IQR is the inter-quartile range, or distance between the first and third quartiles). The lower whisker extends from the hinge to the smallest value at most 1.5 * IQR of the hinge. Data beyond the end of the whiskers are called "outlying" points and are plotted individually. Density plots were generated with the ‘geom_flat_violin’ option from the “raincloudplots” package. Estimation of densities and the resulting number of peaks were done using the default settings of the ‘density()’ function in the base R “stats” package using a Gaussian smoothing kernel which defaults to 0.9 times the minimum of the standard deviation and the interquartile range divided by 1.34 times the sample size to the negative one-fifth power (Silverman's ‘rule of thumb’); unless the quartiles coincide, when a positive result will be guaranteed. Clustering heatmaps were generated using the “ComplexHeatmap” package. Crosshair plots depict the median and standard deviations. Plots depicting linear associations were generated with ggplot's ‘geom_point()’ function and where linear relations are reported include shaded regions indicating the 95% confidence intervals of that linear relation. Linear regression was performed using the “lm” function in the base “stats” package, as well as the “lmerTest” package for mixed-effects modelling. Student’s T-tests were performed using the “t.test” function in the base “stats” package (two-sided, unless otherwise reported). The “ggstatsplot” package was used for the model generalisability analyses to report robust correlation values. Cohen’s d effect sizes were calculated using the “effsize” package. A description of the FreeSurfer version and processing pipeline can be found in SI18 (mainly FreeSurfer 6.0.1 unless stated otherwise).

For manuscripts utilizing custom algorithms or software that are central to the research but not yet described in published literature, software must be made available to editors and reviewers. We strongly encourage code deposition in a community repository (e.g. GitHub). See the Nature Portfolio guidelines for submitting code & software for further information.

Data

Policy information about availability of data

All manuscripts must include a data availability statement. This statement should provide the following information, where applicable:

- Accession codes, unique identifiers, or web links for publicly available datasets
- A description of any restrictions on data availability
- For clinical datasets or third party data, please ensure that the statement adheres to our policy

Model parameters and out-of-sample centile scores are available at www.brainchart.io and on https://github.com/brainchart/Lifespan. Summary statistics are available in the Supplementary Tables (ST1-8). Links to open and semi-open datasets are also listed on https://github.com/brainchart/Lifespan. Availability of other MRI datasets aggregated here is through application procedures individually managed at the discretion of each primary study, with additional information provided in ST1.1 and SI19.

Field-specific reporting

Please select the one below that is the best fit for your research. If you are not sure, read the appropriate sections before making your selection.

☒ Life sciences ☐ Behavioural & social sciences ☐ Ecological, evolutionary & environmental sciences

For a reference copy of the document with all sections, see nature.com/documents/nr-reporting-summary-flat.pdf

Life sciences study design

All studies must disclose on these points even when the disclosure is negative.

Sample size	No a-priori sample size was calculated, but we used the largest sample of neuroimaging data reported to date and conduct multiple sensitivity analyses in addition to the built ML optimisation of our models to ensure data was robust.
Data exclusions	Exclusion criteria for each dataset at input stage was determined by collecting sites and studies and are listed in the supplementary materials (SI19) where each dataset is described and where relevant. Missing demographic data or failure in image processing (either due to technical problems with the data or other artefacts) was a secondary reason for exclusion.
Replication	Reproducibility of findings was ensured by extensive sensitivity and bootstrapping analysis, simulation of model parameters, evaluation of optimal model parameters, validation using iterative leave-one-out analysis, and validation against known growth charts derived from other modalities.
Randomization	For our bootstrapping we used random sampling maintaining dataset ratios as described in the supplementary methods. For pairwise comparisons between control and clinical cohorts we used permutation tests that randomly reshuffle case and control labels to generate 10,000 null distributions.
Blinding	Blinding was not possible, but also not applicable for establishing growth trajectories, furthermore all analyses were conducted in a data driven manner

Reporting for specific materials, systems and methods

We require information from authors about some types of materials, experimental systems and methods used in many studies. Here, indicate whether each material, system or method listed is relevant to your study. If you are not sure if a list item applies to your research, read the appropriate section before selecting a response.

Materials & experimental systems

n/a	Involved in the study	
☒	☐	Antibodies
☒	☐	Eukaryotic cell lines
☒	☐	Palaeontology and archaeology
☒	☐	Animals and other organisms
☐	☒	Human research participants
☒	☐	Clinical data
☒	☐	Dual use research of concern

Methods

n/a	Involved in the study	
☒	☐	ChIP-seq
☒	☐	Flow cytometry
☐	☒	MRI-based neuroimaging

Human research participants

Policy information about studies involving human research participants

Population characteristics	Population characteristics are listed in supplementary tables 1.1-1.48. For the analysis age and sex were included in our models. Diagnosis was provided for each dataset individually and procedures for obtaining these were described in the description of each individual dataset.
Recruitment	All analyses in the present manuscript were based on existing data. Recruitment for each existing dataset is described in the supplementary description for each dataset see SI19.
Ethics oversight	The project received IRB exemption from CHOP and ethical approval from the Psychology Research Ethics Committee at the University of Cambridge. All contributing datasets already contained their own respective ethical oversight and therefore both committees concluded no additional ethical approval was required. The following statement has been added to the methods section:<br>The research was reviewed by the Cambridge Psychology Research Ethics Committee (PRE.2020.104) and The Children's Hospital of Philadelphia's Institutional Review Board (IRB 20-017874) and deemed not to require PRE or IRB oversight as it consists of secondary analysis of de-identified primary datasets. Informed consent of participants (or their guardians) in primary studies is referenced in supplementary information [SI] 19 and supplementary table [ST] 1.

Note that full information on the approval of the study protocol must also be provided in the manuscript.

Magnetic resonance imaging

Experimental design

Design type	Structural MRI
Design specifications	No specific experimental setup was used
Behavioral performance measures	No behavioural measures are included

Acquisition

Imaging type(s)	Structural, mainly T1 and/or T2 weighted imaging, variations of each dataset are listed in detail in supplementary table 1.1
Field strength	Varying (description in each dataset description and supplementary table 1.1 under the column "Field Strength")
Sequence & imaging parameters	Varying (description in each dataset description and supplementary table 1.1)
Area of acquisition	Whole brain

Diffusion MRI ☐ Used ☒ Not used

Preprocessing

Preprocessing software	Varying (description in each dataset description) but mainly based on Freesurfer recon-all
Normalization	Varying (description in each dataset description) but mainly based on Freesurfer recon-all
Normalization template	Varying (description in each dataset description) but mainly based on Freesurfer recon-all (e.g. fsaverage)
Noise and artifact removal	Varying (description in each dataset description) but mainly based on Freesurfer recon-all
Volume censoring	None

Statistical modeling & inference

Model type and settings	We used generalised additive models for location scale and shape (GAMLSS) to estimate cross-sectional normative age-related trends.
Effect(s) tested	We modelled growth trajectories and generated individual centile scores from these growth charts

Specify type of analysis: ☒ Whole brain ☐ ROI-based ☐ Both

Statistic type for inference (See Eklund et al. 2016)	Not applicable
Correction	For any pairwise comparisons we used Monte-Carlo permutation tests and report all Benjamini-Hochberg FDR corrected values in addition to Cohens d effect sizes

Models & analysis

n/a	Involved in the study
☒	☐ Functional and/or effective connectivity
☒	☐ Graph analysis
☐	☒ Multivariate modeling or predictive analysis

Multivariate modeling and predictive analysis	We used generalised additive models for location scale and shape (GAMLSS) to estimate cross-sectional normative age-related trends. Including study, sex and processing pipeline as random effects in higher order polynomial models.
